# The effect of neuromuscular training on an edentulous patient with unstable mandibular position and uncoordinated mandibular movements: a clinical report

**DOI:** 10.1186/s12903-025-05952-0

**Published:** 2025-04-15

**Authors:** Hong Zhang, Shuying Hu, Yue Li, Xiaojing Yang, Mei Rui, Xiaoping Luo

**Affiliations:** Nanjing Stomatological Hospital, Affiliated Hospital of Medical School, Institute of Stomatology, Nanjing University, Nanjing, 210008 China

**Keywords:** Case report, Complete denture, Neuromuscular training, Therapeutic complete denture, Mandibular position, Mandibular movement, Gothic arch tracing, Tissue conditioner

## Abstract

**Background:**

The edentulous patients with unstable mandibular position (MP) and uncoordinated mandibular movement (MM) usually complain the poor therapeutic effect when conventional complete dentures (CCDs) are delivered. This case report aims to observe whether neuromuscular training (NT) using therapeutic complete dentures (TCDs) can improve the MP and MM, thereby promoting the effect of the definitive dentures.

**Case presentation:**

NT was conducted using TCDs for an edentulous patient with unstable MP and uncoordinated MM. After a period of masticatory exercises, the patient’s MP and MM improved. Finally, the definitive dentures were delivered, achieving satisfactory results.

**Conclusion:**

NT with TCDs can improve unstable MP and uncoordinated MM, leading to successful rehabilitation in edentulous patient.

## Background

Accurate mandibular position (MP) and coordinated mandibular movements (MM) are critical for functional rehabilitation in edentulous patients [1, 2]. However, unstable MP and uncoordinated MM are increasingly common in edentulous patients, often caused by poor chewing habits and some systemic conditions such as cerebral palsy, Bell’s palsy, and Parkinson’s disease [3–5]. These conditions can lead to unstable occlusion, making chewing difficult and increasing occlusal interference [6]. 

Even when centric occlusion is established based on the apex point of gothic arch tracing (GAT) trajectory, these patients frequently complain of poor retention, bad stability, pain, and recurrent mucosal ulcers when using conventional complete dentures (CCDs) [7]. Furthermore, occlusal trauma due to unstable MP and uncoordinated MM is especially harmful in implant-supported complete dentures, leading to complications like fractured artificial teeth, cracked bridge frameworks, peri-implantitis, implant loosening, and, in more serious cases, temporomandibular joint dysfunction and orofacial pain [8]. 

Therapeutic complete dentures (TCDs) with flat occlusal tables in the mandibular denture allow unrestricted voluntary movements, promoting neuromuscular adaptation by reducing occlusal interference and enabling repetitive masticatory exercises to restore coordinated muscle function. These dentures have shown promise in improving treatment effect for edentulous patients with complex conditions. Previous reports have demonstrated the successful stabilization of complete dentures in patients with cerebral palsy, Bell’s palsy, and Parkinson’s disease using TCDs [3–5]. It has been suggested that when flat table treatment dentures are used, the oral mucosa provides sensory feedback regarding vertical stop and bite force, leading to increased activation of the masseter muscle [3]. However, detailed protocols for therapeutic procedures, including specific methods, duration, and efficacy, are not well documented, and the underlying mechanism of treatment remains unclear.

This clinical report presents a detailed protocol for neuromuscular training (NT) in an elderly edentulous male patient with an unstable MP and uncoordinated MM. The effectiveness of NT was systematically evaluated, and the final definitive dentures achieved satisfactory results. Through this case report, we aim to provide a reproducible and practical therapeutic approach for managing similar patients.

## Case presentation

An 82-year-old male patient presented at Nanjing Stomatological Hospital, Affiliated Hospital of Medical School, Institute of Stomatology, Nanjing University (Nanjing, China), complaining of tooth loss and recovery his chewing ability. He reported a decade-long history of unsuccessful attempts to use removable partial dentures (RPDs) due to their ineffectiveness and pain during mastication, which led him to rely solely on his right premolars for chewing. However, three months ago, he lost his right mandibular premolars, rendering him unable to chew any food, significantly affecting his nutritional intake and overall quality of life. The patient was in good general health, with no history of severe systemic diseases (e.g., hypertension, diabetes, or cardiovascular disorders), psychiatric conditions, or neurological deficits (e.g., Parkinson’s disease or stroke). He also reported no pain in the temporomandibular joints and denied smoking, alcohol consumption, or any parafunctional habits. Clinical examination revealed four natural teeth without obvious mobility in the maxillary arch and edentulous mandibular arch. The mandibular residual ridge was well-rounded (Atwood Type I), and the oral mucosa was well-keratinized. A treatment plan proposing an implant-supported overdenture for the mandible and a RPD for the maxilla was presented. However, the patient opted for a removable prosthesis for both arches, avoiding surgery.

**Fig. 1:**
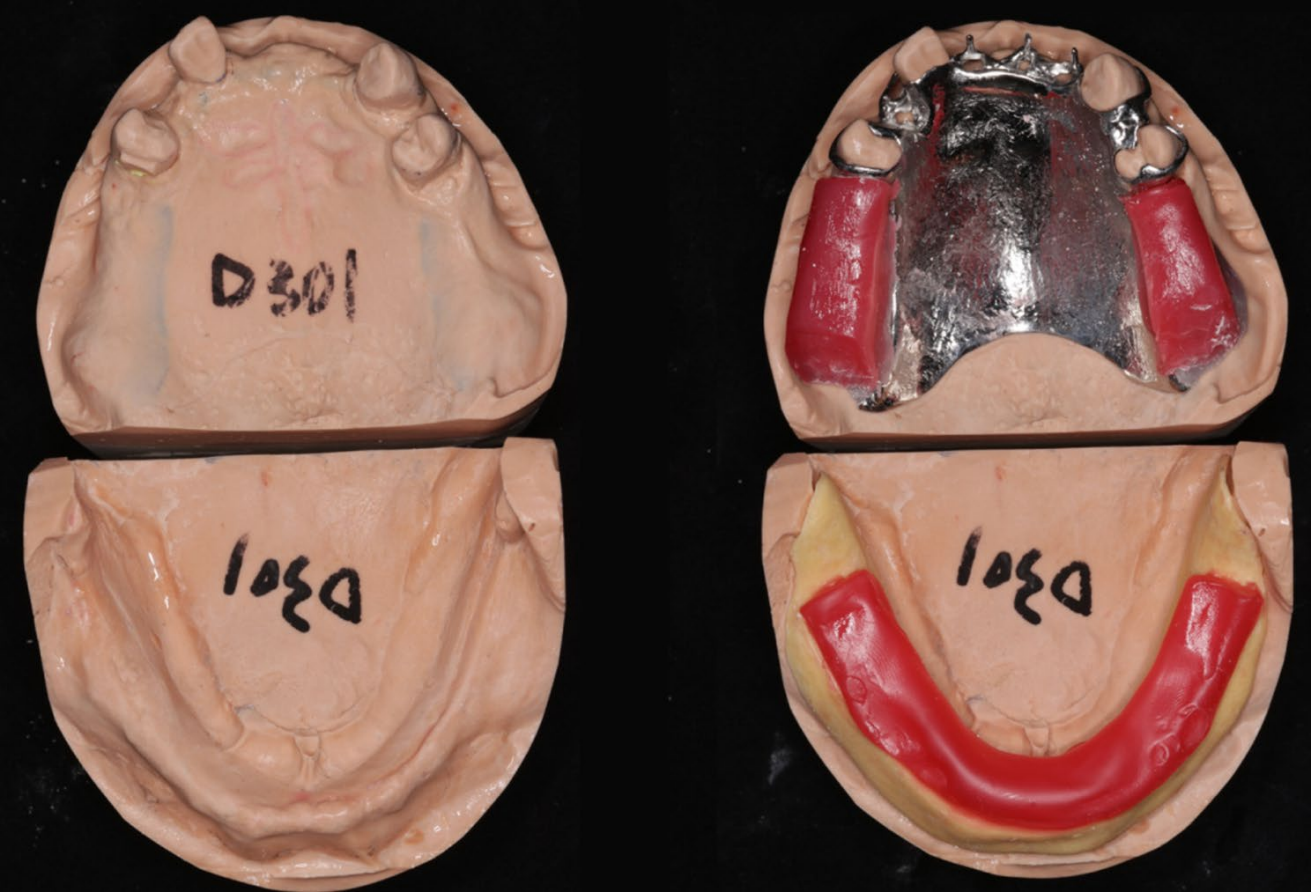
The left side showed the maxillary master cast and the mandibular preliminary cast, while the right side displayed the maxillary metallic framework and the mandibular occlusion registration plate

**Fig. 2:**
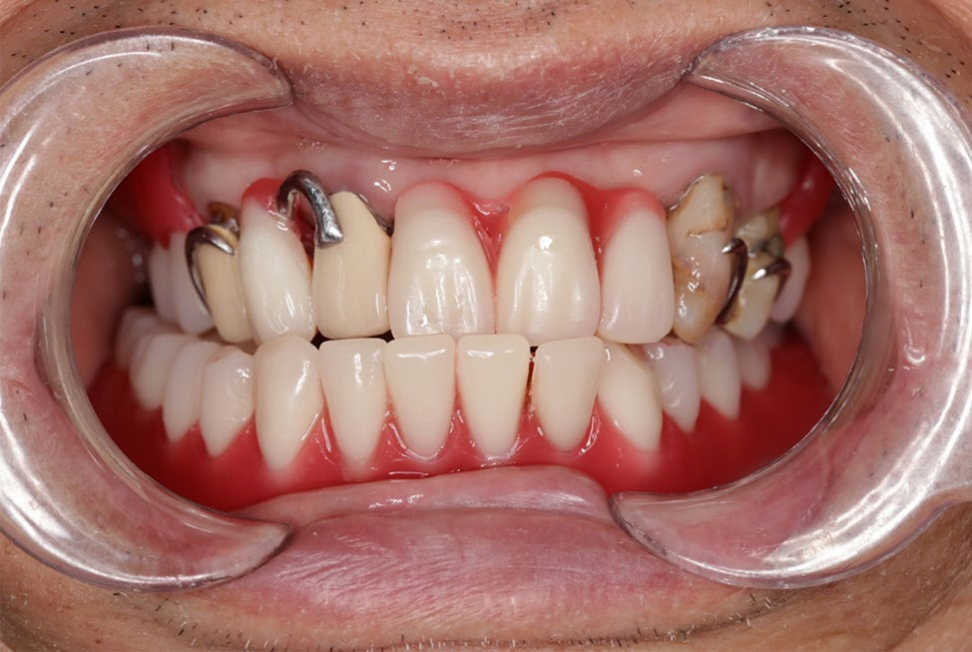
At the first try-in, the patient’s mandible exhibited a habitual forward and rightward shift during mouth closure

**Fig. 3:**
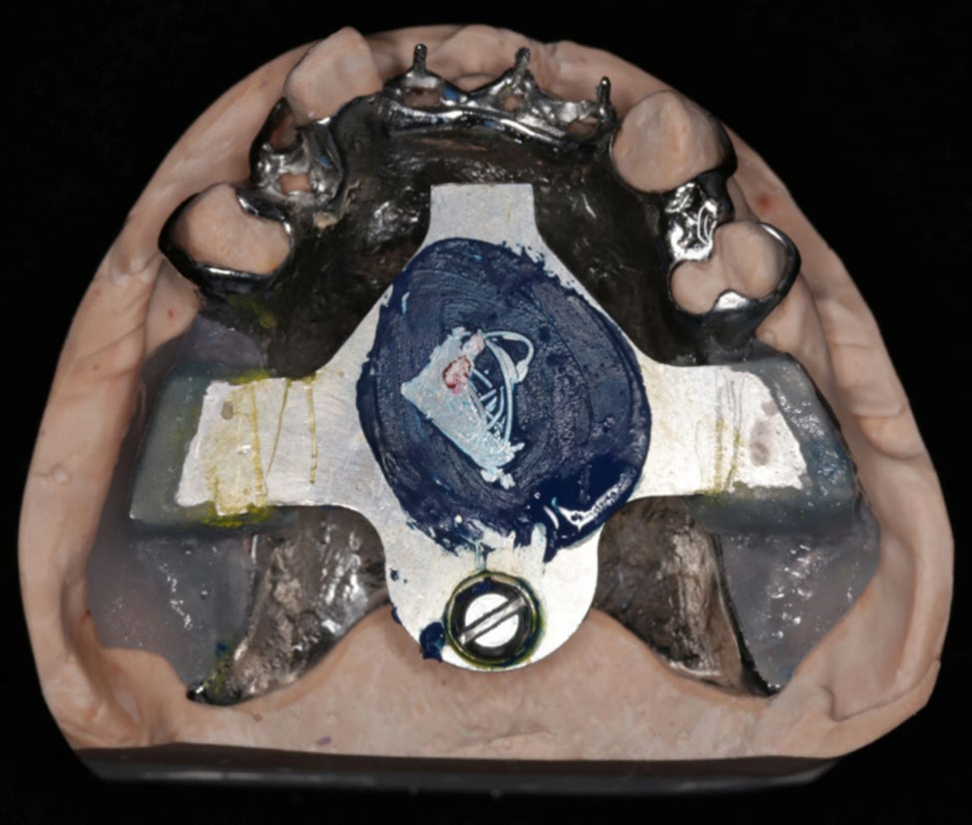
Before NT, GAT trajectory showed scattered tapping points far from the apex point, along with a noticeable deficiency in left MM paths

Routine procedures were initiated for both the maxillary and mandibular dentures. The maxillary master cast and mandibular preliminary cast were made, and the metallic framework for the maxillary RPD and the mandibular occlusion registration plate were fabricated (Fig. 1). Occlusion registration was recorded using check-bite method with wax rims. However, at the first try-in, the patient’s mandible exhibited a habitual forward and rightward shift during mouth closure (Fig. 2). To confirm the centric relationship, GAT (Gnathometer M, Ivoclar Vivadent AG), mounted on the maxillary metallic framework and the mandibular occlusion plate, was used. The patient was seated upright with proper head support, and instructed to perform protrusive, retrusive and lateral jaw movements. Then the rapid and continuous tapping movements were also instructed. GAT trajectory showed scattered tapping points far from the apex point, along with a noticeable deficiency in left MM paths (Fig. 3).

Given the negative impact of the unstable MP and uncoordinated MM, NT with TCDs before the definitive dentures delivered was recommended. After a detailed explanation, the patient consented to the treatment protocol.

**Fig. 4:**
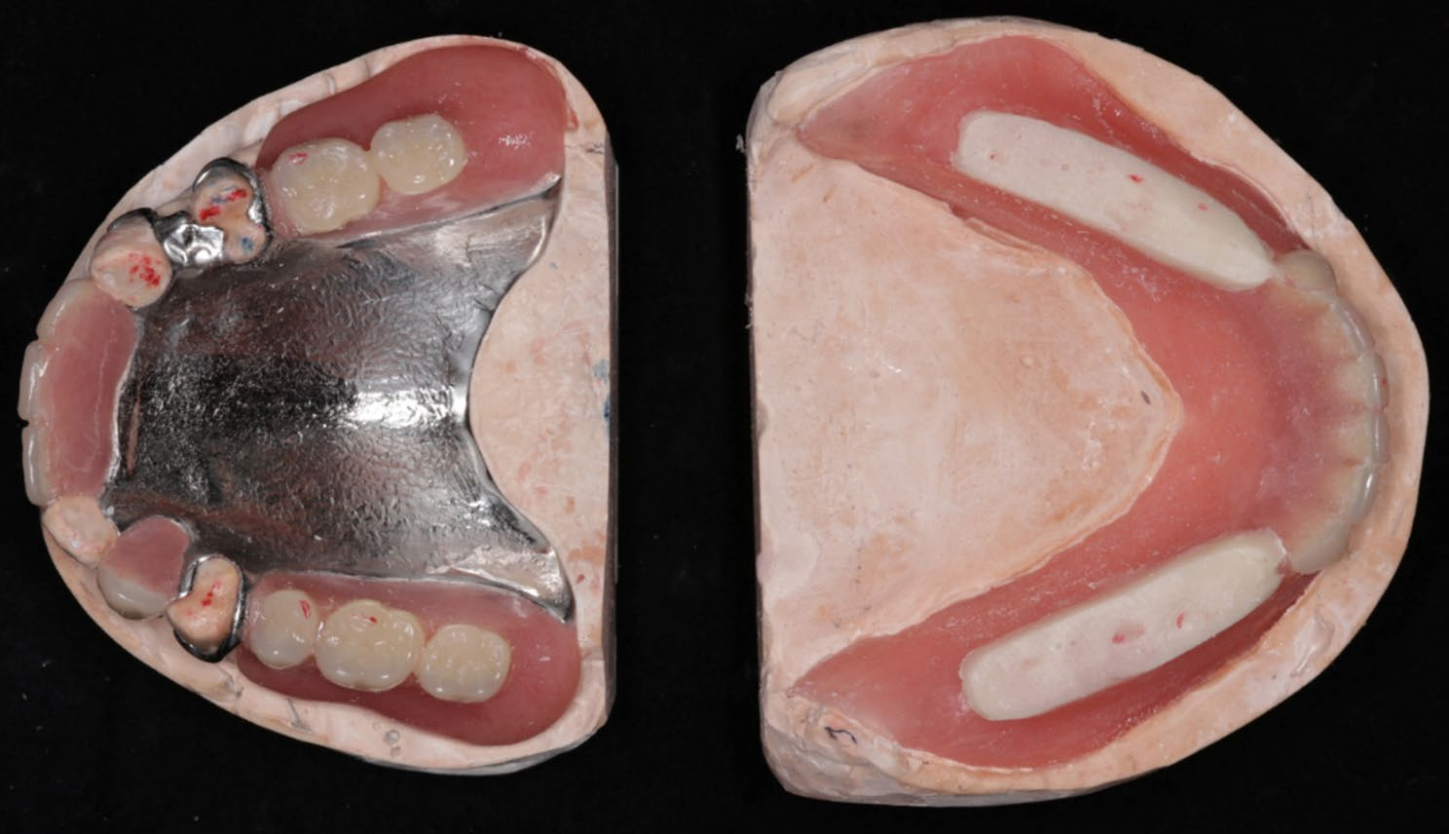
The point-flat contacts without cusp-fossa interlocks between the maxillary functional cusps and mandibular flat tables

The horizontal jaw relationship was re-established at the apex point based on GAT trajectory, and the occlusal plane was transferred with a face bow. The maxillary RPD was fabricated following standard procedures. The mandibular posterior artificial teeth were instead of flat occlusal tables, paralleled to the occlusal plane. They were made of a mixture of auto-polymerizing acrylic resin and zinc oxide powder in a 1:1 ratio, allowing only the maxillary functional cusps to contact them, without any cusp-fossa interlocks (Fig. 4). The mucosal surface of the mandibular denture base was lined with tissue conditioner (TC) (Coe-Comfort, GC Ltd.).

After the delivery of the TCDs, the patient was instructed to perform NT through masticatory exercises at least three times a day—during breakfast, lunch, and dinner— with each session lasting a minimum of 30 min. The progression of food texture was carefully planned, transitioning gradually from soft to normal foods. Weeks 1–2: Soft foods such as noodles, porridge, steamed eggs, and tofu were recommended to minimize mucosal pressure and allow initial adaptation. Weeks 3–4: Semi-solid foods, including rice, fish, tomatoes, scrambled eggs, and ground meat, were introduced to gradually increase the masticatory load. Weeks 5–8: Solid foods, such as nuts, raw cucumber, braised pork, and stewed chicken, were incorporated to simulate natural chewing demands and further enhance neuromuscular coordination. Advancement to the next food texture level was permitted only if the patient reported no mucosal pain or ulceration after chewing, ensuring a comfortable and progressive adaptation process. Follow-up visits were scheduled every two weeks to adjust the occlusal contacts and TC layer. Initially, unbalanced indentations were observed on the mandibular flat tables, and these, along with any obvious cusp-fossa interlocks, were adjusted to balanced point-flat contacts. Any flushed areas on TC layer were ground down and relined with new TC. GAT trajectory was observed at each visit to monitor the changes of MP and MM.

**Fig. 5:**
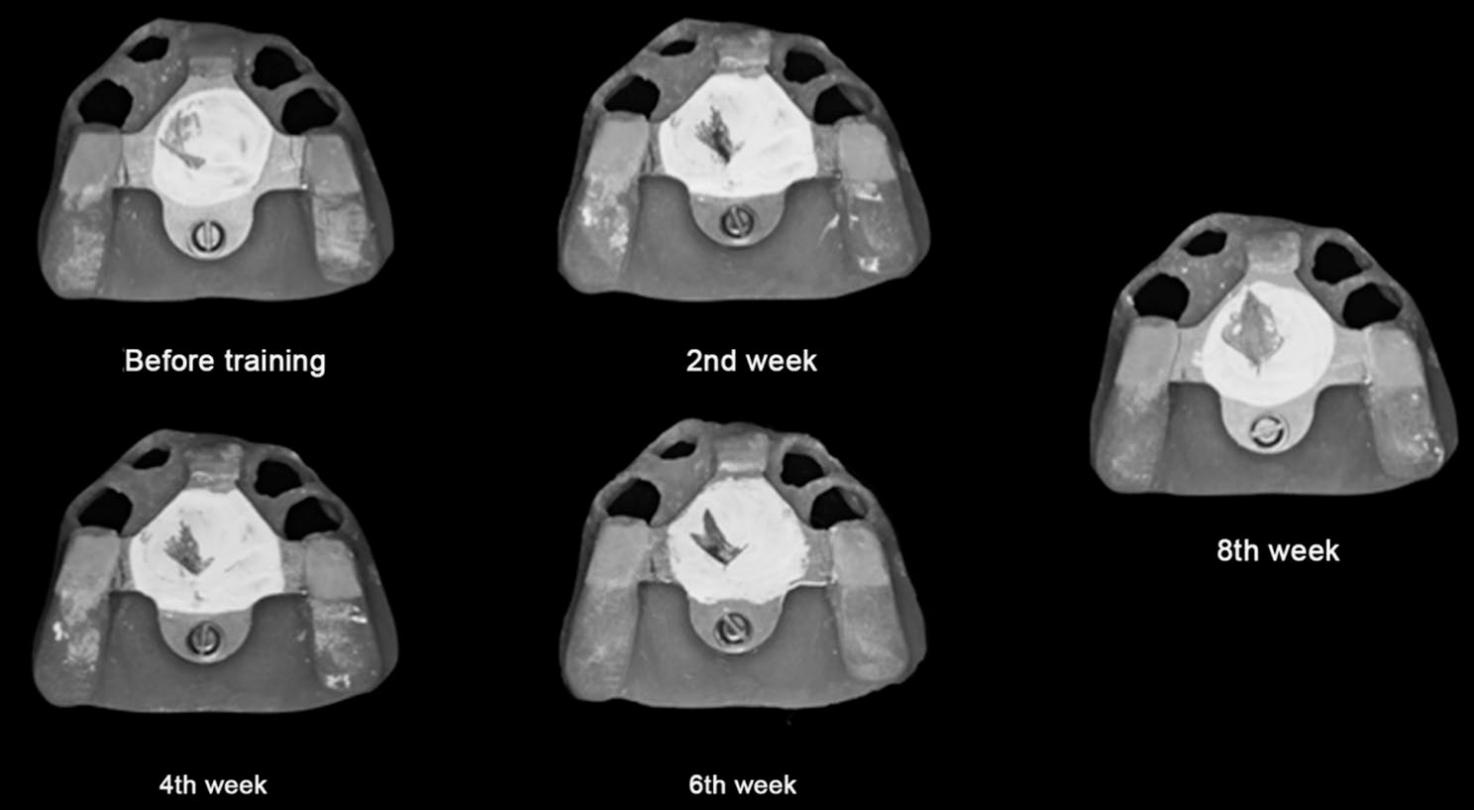
GAT trajectories indicated an improvement in mandibular movement paths during NT

**Fig. 6:**
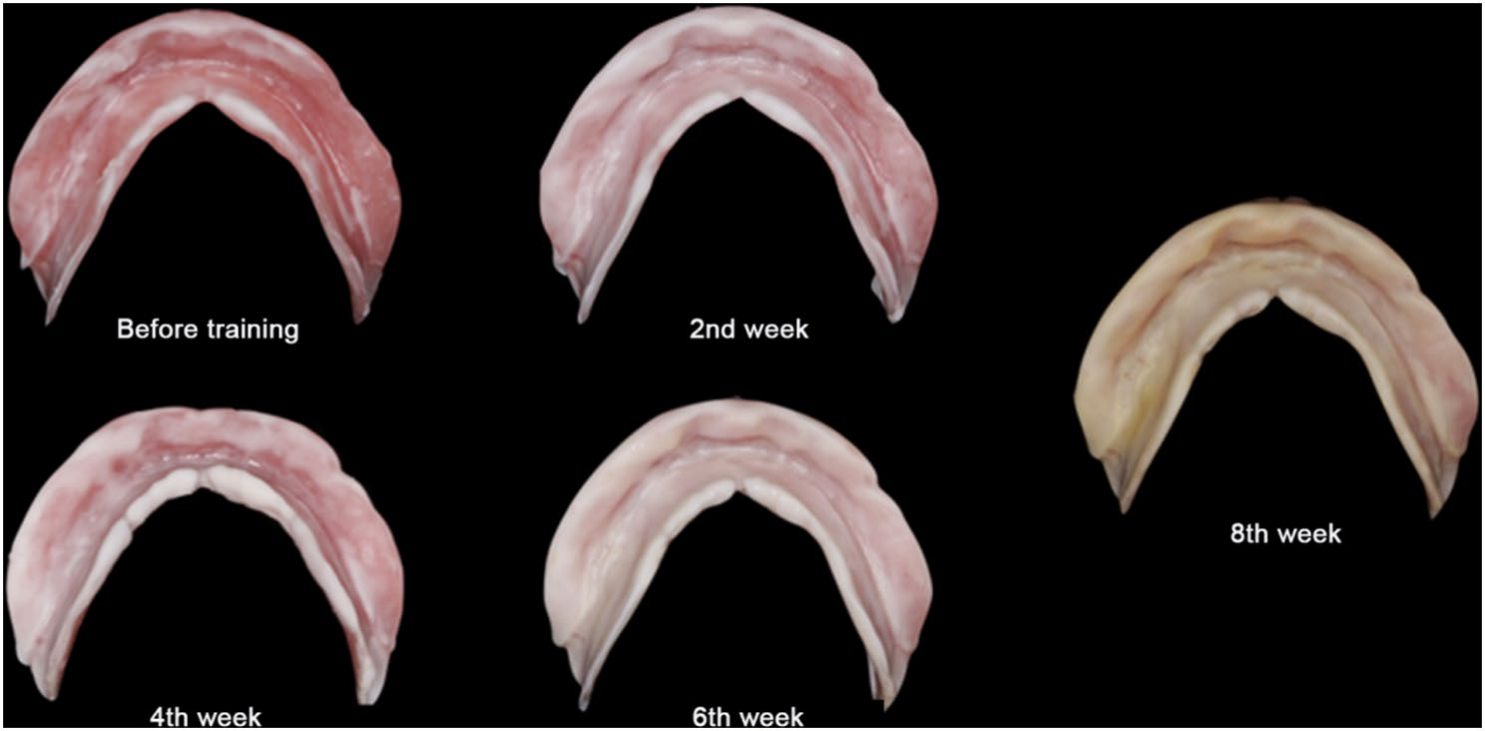
TC layers on the mucosa surface of the denture base became more uniform

**Fig. 7:**
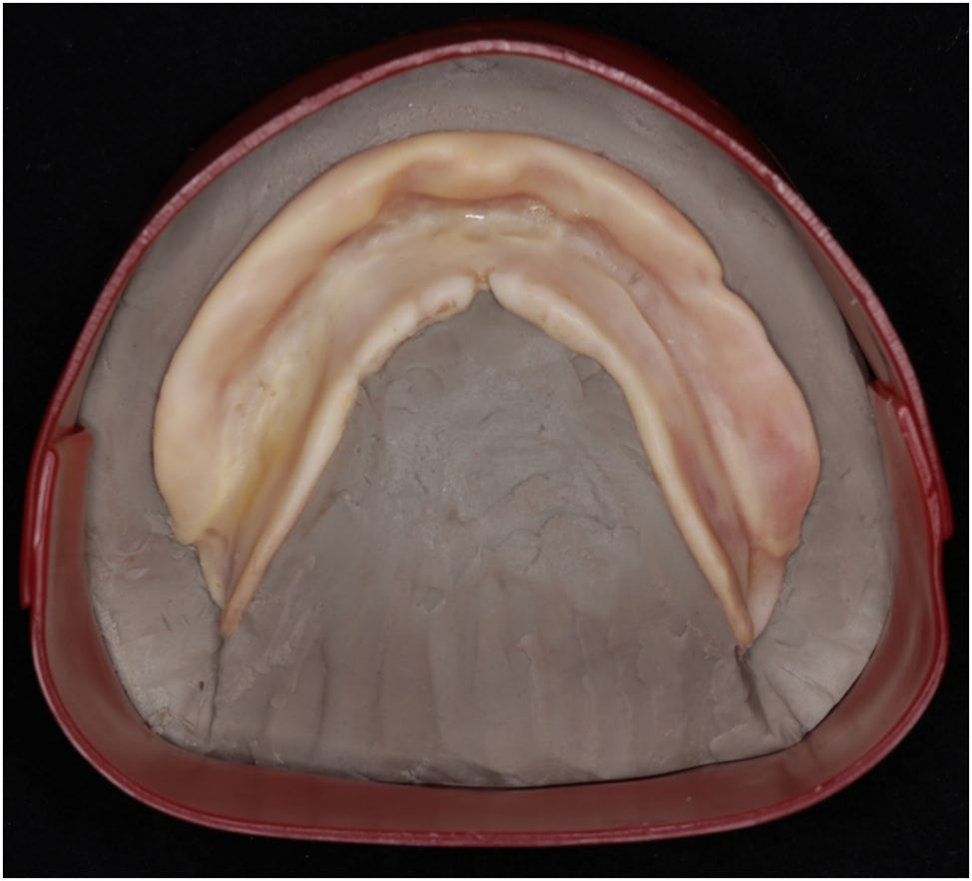
The ultimate TC layer served as the dynamic functional impression for the definitive mandibular dentures

**Fig. 8:**
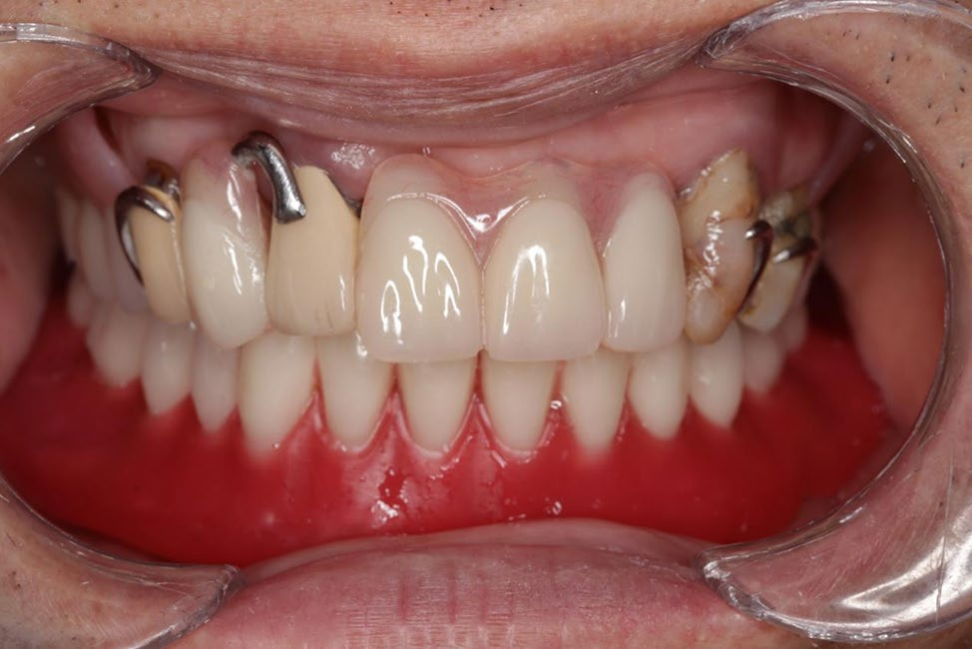
The final try-in dentures were in the mouth

**Fig. 9:**
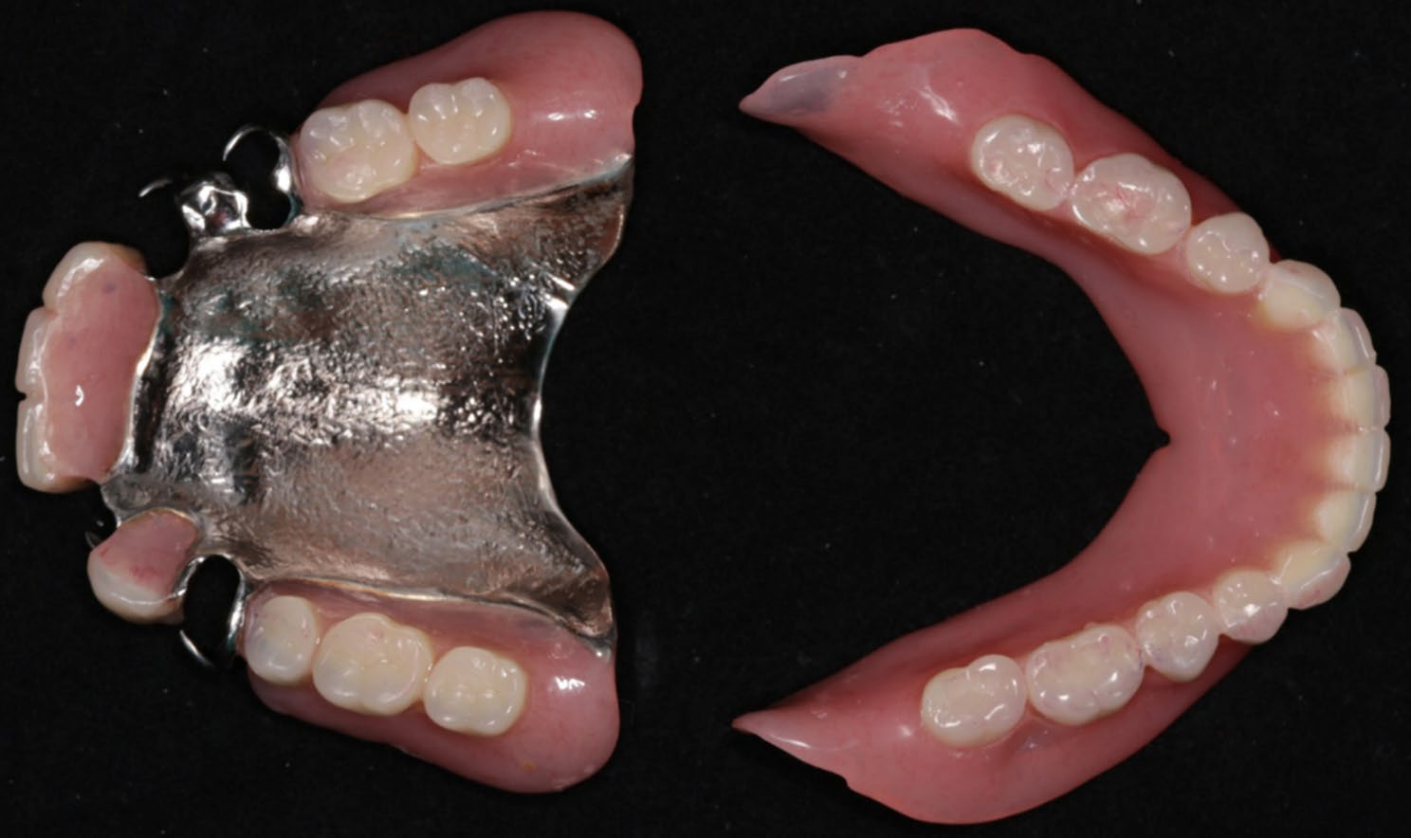
The definitive maxillary and mandibular dentures were delivered

At the eighth-week follow-up, GAT trajectory returned to a typical arrowhead shape (Fig. 5), with clear indentations on the mandibular flat tables and no flushed areas on TC layer (Fig. 6). At this time point, NT was deemed complete, and the definitive mandibular denture was fabricated. The relationship between the maxilla and mandible was recorded using silicone bite registration material (Virtual CADbite Registration, Ivoclar Vivadent AG), and TC layer was used as a dynamic functional impression (Fig. 7). The mandible final try-in denture was made and evaluated in the patient’s mouth (Fig. 8). Finally, the definitive mandibular complete denture was fabricated (Fig. 9).

The patient’s satisfaction regarding retention, stability, chewing ability and phonetics was evaluated using a Visual Analog Scale (VAS) method at each follow-up visit, and 1 day, 1 month, 6 months, and 1 year after the definitive dentures were delivered (see Table 1).

**Table 1:** The VAS grades (0–10 scale) of the retention, stability, chewing ability and phonetics

Time points	Retention	Stability	Chewing ability	Phonetics
Before training	3	2	1	4
2 weeks training	5	3	2	6
4 weeks training	6	5	5	8
6 weeks training	8	8	8	10
8 weeks training	10	9	8	10
1 day after delivered	10	9	8	10
1month after delivered	10	9	8	10
6 months after delivered	10	10	9	10
1 year after delivered	10	10	10	10

## Discussion and conclusions

In natural teeth, the signals of occlusal contacts are perceived by mechanoreceptors within the periodontium, which activate sensorimotor circuits and provide feedback to promote rhythmic jaw movements [9]. However, in edentulous patients, these signals are received by the mucosa beneath the denture base and the perioral muscles [10]. In this case, due to long-term tooth loss and ill-fitting dentures, the input signals from the oral mucosa and muscles are significantly reduced and inaccurate, leading to neuromuscular dysfunction. At the first try-in, the patient showed unstable MP and the patient’s mandible exhibited a habitual forward and rightward shift during mouth closure.

To resolve the poor chewing habit, normal neuromuscular function must be rebuild. Previous studies have demonstrated that repetitive motor training, such as tooth clenching [11], hold-and-split training for anterior teeth [12], and tongue movements [13, 14], can induce cortical plasticity of the jaw muscles, optimizing muscle function. In this case, we suspected that the mechanism of TCDs treatment is plasticity in the motor cortex of the masticatory muscles induced by repeated masticatory exercises. Only with restored neuromuscular function can dentures serve as effective tools for regaining optimal chewing function.

As an appliance of NT, TCDs are characterized by point-flat contacts between the functional cusps of the maxillary denture and the flat tables of the mandibular denture, allowing voluntary mandibular movements without restriction [4]. To maintain voluntary movement, regular occlusal adjustments are crucial at each follow-up visit. In this case, after a period of masticatory exercises, the indentations became more balanced, indicating improved MM.

GAT is a simple and effective technique for determining the centric relationship between maxilla and mandible (apex point) [15]. By analyzing the relationship between the muscular contact position (tap point) and the apex point, as well as the lateral mandibular movement paths, neuromuscular function can be inferred [16, 17]. In this case, after a period of NT, GAT showed an improved trajectory, finally approaching the typical arrowhead shape, indicating improvements in MP and MM. Instead of relying on clinician’s experience, the typical arrowhead shape serves as a clear indicator for terminating of TN. Therefore, using GAT to guide the therapeutic process is more precise and practical, making it a valuable approach to promote in future clinical practice.

TC is a viscous gel with viscoelastic properties [18] that can help restore inflamed oral mucosa beneath the denture base, record dynamic functional impressions, adjust occlusal force distribution, and alleviate pain [19–21]. In this case, changes in TC layer during treatment reflected the effects of NT, and its final uniformity indicated improvements in MP and MM. However, due to ethanol evaporation and the aging of plasticizers, TC layer gradually loses its viscoelastic properties, requiring reapplication every two weeks [22]. Ultimately, TC layer served as the dynamic functional impression for the definitive mandibular denture [23]. 

Although the satisfaction questionnaire is a subjective evaluation method, it is widely used in the bio-psycho-social model of medical care to assess treatment effect in edentulous patients [24]. In this case, the patient’s subjective evaluations aligned with the objective results from GAT trajectory and TC performance, confirming the effectiveness of NT with TCDs. Throughout NT period and after the delivery of the definitive dentures, the patient reported none of the pain he had experienced with traditional dentures.

While this case demonstrates the effectiveness of TCDs, further research is needed to clarify the effects of NT on the brain, masticatory muscles, and condylar position by transcranial magnetic stimulation (TMS), electromyography (EMG), or cone beam computed tomography (CBCT).

Neuromuscular training with therapeutic complete dentures can improve unstable mandibular positions and uncoordinated mandibular movements, leading to successful rehabilitation in edentulous patients.

## Data Availability

All data generated or analyzed during this study are included in this published article.

## Abbreviations

TCDs: Therapeutic Complete Dentures
CCD: Conventional Complete denture
TC: Tissue conditioner
NT: Neuromuscular training
RPD: Removable Partial Dentures
GAT: Gothic Arch Tracing
MP: Mandibular position
MM: Mandibular movement
VAS: Visual Analog Scale
TMS: Transcranial Magnetic Stimulation
EMG: Electromyography
CBCT: Cone Beam Computed Tomography

## References

[1] Li Z, Xia Y, Chen K, Zhao H, Liu Y. Maintenance of the maxillomandibular position with digital workflow in oral rehabilitation: A technical note. Int J Prosthodont. 2018;31:280–2. 29723325 10.11607/ijp.5663

[2] Santos IC, Tavares JM, Mendes JG, Paulo MP. Acquisition and analysis of 3D mandibular movement using a device based on electromagnetic sensors and a neural network. J Med Eng Technol. 2009;33:437–41. 19401907 10.1080/09593980902886354

[3] Inada M, Yamazaki T, Shinozuka O, Sekiguchi G, Tamamori Y, Ohyama T. Complete denture treatments for a cerebral palsy patient by using a treatment denture. A case report. J Med Dent Sci. 2002;49:171–7. 12641388

[4] Rajapur A, Mitra N, Prakash VJ, Rah SA, Thumar S. Prosthodontic rehabilitation of patients with Bell’s palsy: our experience. J Int Oral Health. 2015;7:77–81. 26668488 PMC4672843

[5] Haralur SB. Clinical strategies for complete denture rehabilitation in a patient with Parkinson disease and reduced neuromuscular control. Case Rep Dent. 2015;2015:352878. 25737785 10.1155/2015/352878 PMC4337048

[6] Ohmichi H. Case report of a complete denture wearer in whom masticatory function improved by correcting horizontal maxillomandibular relationship. Nihon Hotetsu Shika Gakkai Zasshi. 2008;52:236–9. 18467799 10.2186/jjps.52.236

[7] Kutkut A, Bertoli E, Frazer R, Pinto-Sinai G, Fuentealba Hidalgo R, Studts J. A systematic review of studies comparing conventional complete denture and implant retained overdenture. J Prosthodont Res. 2018;62:1–9. 28666845 10.1016/j.jpor.2017.06.004

[8] Caloss R, Al-Arab M, Finn RA, Throckmorton GS. The effect of denture stability on bite force and muscular effort. J Oral Rehabil. 2011;38:434–9. 21050259 10.1111/j.1365-2842.2010.02169.x

[9] Kumar A, Kothari M, Grigoriadis A, Trulsson M, Svensson P. Bite or brain: implication of sensorimotor regulation and neuroplasticity in oral rehabilitation procedures. J Oral Rehabil. 2018;45:323–33. 29314189 10.1111/joor.12603

[10] Ito N, Kimoto S, Kawai Y. Does wearing dentures change sensory nerve responses under the denture base? Gerodontology. 2014;31:63–7. 23278139 10.1111/ger.12006

[11] Iida T, Overgaard A, Komiyama O, Weibull A, Baad-Hansen L, Kawara M, Sundgren PC, List T, Svensson P. Analysis of brain and muscle activity during low-level tooth clenching–a feasibility study with a novel biting device. J Oral Rehabil. 2014;41:93–100. 24393147 10.1111/joor.12128

[12] Zhang H, Kumar A, Kothari M, Luo X, Trulsson M, Svensson KG, Svensson P. Can short-term oral fine motor training affect precision of task performance and induce cortical plasticity of the jaw muscles? Exp Brain Res. 2016;234:1935–43. 26914481 10.1007/s00221-016-4598-4

[13] Matsuzaki S, Shimada A, Tanaka J, Kothari M, Castrillon E, Iida T, Svensson P. Effect of mandibular advancement device on plasticity in corticomotor control of tongue and jaw muscles. J Clin Sleep Med. 2021;17:1805–13. 33904391 10.5664/jcsm.9284 PMC8636349

[14] Iida T, Komoda Y, Kothari M, Sekihata S, Komiyama O, Sessle B, Svensson P. Combination of jaw and tongue movement training influences neuroplasticity of corticomotor pathways in humans. Exp Brain Res. 2019;237:2559–71. 31346648 10.1007/s00221-019-05610-2

[15] Potdukhe SS, Iyer JM, Nadgere JB. Evaluation of accuracy between extraoral Gothic arch tracing and various other methods assessing horizontal condylar guidance angle in completely edentulous patients: A systematic review and meta-analysis. J Indian Prosthodont Soc. 2023;23:322–34. 37861609 10.4103/jips.jips_216_23 PMC10705011

[16] de Sousa Ervolino IC, Goiato MC, de Moraes Melo Neto CL, de Caxias FP, da Silva EVF, Túrcio KHL, Dos Santos DM. Clinical reproducibility of different centric relation recording techniques in edentulous individuals: an observational cross-sectional study. J Prosthodont. 2023;32:497–504. 36573906 10.1111/jopr.13635

[17] de Moraes Melo Neto CL, Dos Santos DM, de Magalhães Bertoz AP, Moreno ALM, Goiato MC. Comparison of techniques for obtaining centric relation based on the reproducibility of the condylar positions in centric relation-A systematic review. Eur J Dent. 2022;16:251–7. 34921385 10.1055/s-0041-1735903 PMC9339936

[18] Kitagawa Y, Yoshida K, Takase K, Valanezhad A, Watanabe I, Kojio K, Murata H. Evaluation of viscoelastic properties, hardness, and glass transition temperature of soft denture liners and tissue conditioner. Odontology. 2020;108:366–75. 31807949 10.1007/s10266-019-00477-9

[19] Emami E, Kabawat M, Rompre PH, Feine JS. Linking evidence to treatment for denture stomatitis: a meta-analysis of randomized controlled trials. J Dent. 2014;42:99–106. 24316341 10.1016/j.jdent.2013.11.021

[20] Mathivanan A, Sayeeganesh N, Raveendran A, Ramya D, Mani J. Treatment of denture stomatitis using modified tissue conditioners: A systematic review. J Pharm Bioallied Sci. 2023;15:S98–100. 37654404 10.4103/jpbs.jpbs_593_22 PMC10466572

[21] Dorocka-Bobkowska B, Medyński D, Pryliński M. Recent advances in tissue conditioners for prosthetic treatment: A review. Adv Clin Exp Med. 2017;26:723–8. 28691420 10.17219/acem/62634

[22] Mikulewicz M, Chojnacka K, Raszewski Z. Comparison of mechanical properties of three tissue conditioners: an evaluation in vitro study. Med (Kaunas). 2023;59:1359. 10.3390/medicina59081359 PMC10456693 37629649

[23] Arora AK, Goyal I, Sehgal M. Comparative evaluation of reproducibility of peripheral tissues produced by different border molding materials in edentulous patients: an in vivo study. J Indian Prosthodont Soc. 2015;15:102–10. 26929495 10.4103/0972-4052.155030 PMC4762307

[24] Herrero F, de Souza RF, Feine JS, Alexander PP, Green AV, Oates TW. The impact of implant-retained overdentures on type-2 diabetic and non-diabetic edentulous patients: satisfaction and quality of life in a prospective cohort study. J Dent. 2022;127:104357. 36351489 10.1016/j.jdent.2022.104357 PMC9691604

